# L’épiphysiolyse fémorale supérieure chez un malade en paralysie cérébrale suite à une crise convulsive

**DOI:** 10.11604/pamj.2018.31.89.6832

**Published:** 2018-10-05

**Authors:** Yassine Nhamoucha, Mohammed Tazi, Hicham Abdellaoui, Othmane Alaoui, Saad Andaloussi, Mohammed Oukhoya, Lamyae Chater, Karima Atarraf, Mounir Arroud, Abderahman Afifi

**Affiliations:** 1 Service de Chirurgie Pédiatrique, CHU Hassan II, Fès, Maroc

**Keywords:** Epiphysiolyse fémorale supérieure, paralysie cérébrale, chirurgie, Slipped capital femoral epiphyses, cerebral palsy, surgery

## Abstract

L'épiphysiolyse fémorale supérieure (EFS) correspond à un glissement de l'épiphyse fémorale supérieure par rapport au col fémoral qui le plus souvent se fait en arrière et en dedans, sous l'effet du poids du corps. Cette affection survient le plus fréquemment lors de la puberté. Nous rapportons le cas d’un enfant atteint de paralysie cérébrale avec spasticité des quatre membres, ce qui représente une entité très rare.

## Abstract (en)

Slipped capital femoral epiphysis (SCFE) is a slippage of the femoral epiphysis (femoral head) on the femoral neck. Femoral epiphysis usually slips backward and inward because of body weight. This disorder mainly occurs during puberty. We report the very rare case of a child with cerebral palsy associated with spasticity of the limbs.

## Introduction

L'épiphysiolyse fémorale supérieure (EFS) correspond à un glissement de l'épiphyse fémorale supérieure par rapport au col fémoral qui le plus souvent se fait en arrière et en dedans, sous l'effet du poids du corps. Les malades atteints de paralysie cérébrale peuvent avoir beaucoup de problèmes au niveau des membres inferieurs et particulièrement au niveau de la hanche, qui peut aller d’une simple dysplasie à une luxation. A notre connaissance un seul cas d’épiphysiolyse fémorale supérieure a été décrit dans la littérature ce qui représente une entité rare. Nous rapportons le cas d’une épiphysiolyse chez un enfant atteint de paralysie cérébrale avec spasticité des quatre membres.

## Patient et observation

**Figure 1:**
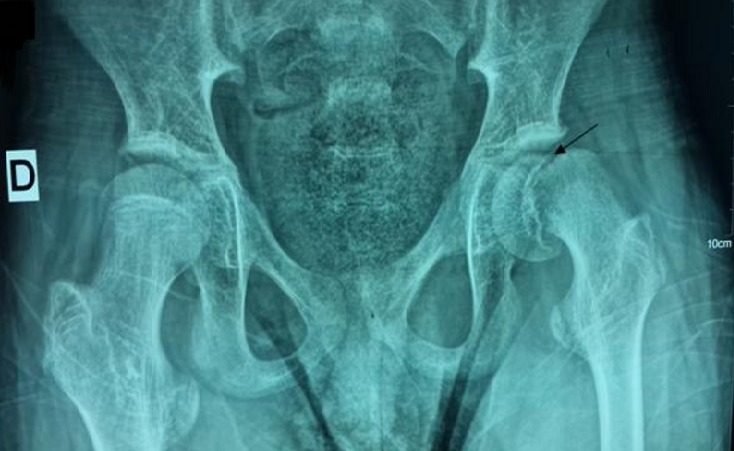
Radiographie du bassin objectivant une épiphysiolyse fémorale supérieure du côté gauche (flèche noire)

**Figure 2:**
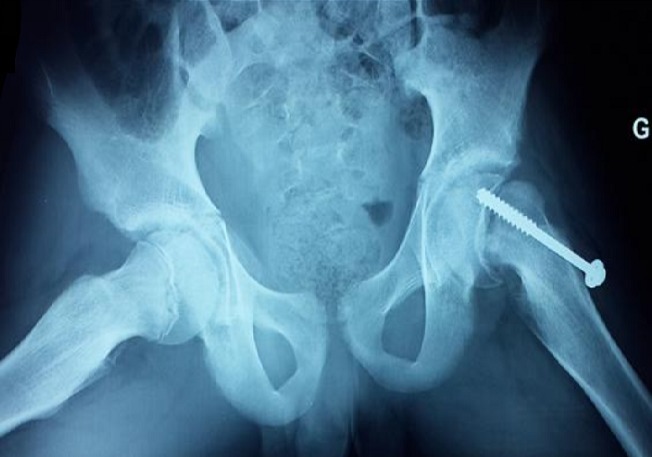
Radiographie du bassin de profil (incidence en grenouille) montrant l’aspect post opératoire après fixation in situ

Il s’agit d’un enfant de 15 ans, ainé d’une fratrie de 3, issu d’un mariage consanguin de 2^ème^ degré. Il est né prématurément à 36 semaines avec une notion de souffrances néonatales, suivi au service de pédiatrie pour retard psychomoteur avec spasticité des quatre membres, admis chez nous pour des douleurs de la hanche évoluant dans un contexte d’apyrexie. Le début de sa symptomatologie remonte à 5 jours avant son admission par l’installation d’une crise convulsive occasionnant chez lui des douleurs de la hanche avec impossibilité à tenir la position assise sans notion de traumatisme ce qui a motivé sa consultation aux urgences pédiatriques pour une prise en charge. L’examen à l’admission trouve un enfant conscient, stable sur le plan hémodynamique et respiratoire, apyrétique, poids à 40 kg avec une spasticité extrême des quatre membres qui sont en flexion. Les deux hanches étaient en adduction fléchis à 80° avec une douleur à la mobilisation de la hanche gauche. La radiographie du bassin de face a objectivé un glissement de l’épiphyse fémorale superieure par rapport à la métaphyse au niveau de la hanche gauche (Figure 1). L’enfant a été opéré à j2 de son admission avec réalisation d’un vissage in situ (Figure 2). Les suites opératoires étaient simples, avec contrôle radiologique satisfaisant. L’enfant est convoqué dans un mois pour contrôle.

## Discussion

La paralysie cérébrale résulte de lésions survenues sur le cerveau en développement du fœtus ou du nourrisson. Ces lésions, non progressives, provoquent un ensemble de troubles permanents du mouvement et de la posture, responsables de limitations d’activités [1]. Il est important de noter que ces désordres ne sont pas causés par des problèmes dans les muscles ou les nerfs, mais par, un développement défectueux ou un dommage aux secteurs moteurs du cerveau, ce qui perturbe la capacité du cerveau à contrôler de manière adéquate le mouvement et la posture [2, 3]. L´épiphysiolyse fémorale supérieure (EFS) se définit comme étant un glissement de l´épiphyse fémorale supérieure par rapport au col fémoral. Certains facteurs de risques épidémiologiques et métaboliques ont été retrouvés [4], avec principalement le surpoids [5, 6]. L’étiologie exacte de l’EFS est toujours inconnue. Il est stipulé qu’il s’agit d’un processus multifactoriel. La littérature part du principe que des facteurs génétiques, biomécaniques et biochimiques jouent un rôle, sans oublier les influences environnementales [7, 8]. Un important facteur biomécanique est le surpoids, dont pratiquement tous les patients souffrent selon la littérature [4]. Pour les malades qui souffrent d’une paralysie cérébrale, les troubles de la hanche sont fréquents, ce qui les expose à un risque plus important de subluxation, de dislocation et de douleur [1]. L’examen clinique est pauvre vu l’état du patient ce qui impose la réalisation d’une radiographie du bassin pour faire le diagnostic [1]. Dans la littérature un cas a été décrit où il a bénéficié d’une tomodensitométrie de la hanche qui a montré un cartilage de croissance déjà fermé, d’où la décision de ne pas opérer ce patient [1].

## Conclusion

L’épiphysiolyse fémorale supérieure reste exceptionnelle chez l’enfant souffrant d’une paralysie cérébrale. A notre connaissance, un seul cas a été rapporté dans la littérature et qui n’a pas été opéré. Pour nous, l’indication opératoire était surtout pour la douleur avec réalisation d’une fixation par vissage.

## Conflits d’intérêts

Les auteurs ne déclarent aucun conflits d’intérêts.
